# The Function and Mechanism of Long Non-coding RNA-ATB in Cancers

**DOI:** 10.3389/fphys.2018.00321

**Published:** 2018-04-10

**Authors:** Huizhong Xiao, Fuyou Zhang, Yifan Zou, Jianfa Li, Yuchen Liu, Weiren Huang

**Affiliations:** ^1^Key Laboratory of Medical Reprogramming Technology, Shenzhen Second People's Hospital, First Affiliated Hospital of Shenzhen University, Shenzhen, China; ^2^University of South China, Hengyang, China; ^3^Shantou University Medical College, Shantou, China; ^4^Guangdong and Shenzhen Key Laboratory of Male Reproductive Medicine and Genetics, Institute of Urology, Peking University Shenzhen Hospital, Shenzhen, PKU-HKUST Medical Center, Shenzhen, China

**Keywords:** long non-coding RNA, ATB, human cancers, therapeutic targets, diagnostic biomarker

## Abstract

Long non-coding RNAs (lncRNAs) are a class of transcriptional RNA molecules with a length of greater than 200 nucleotides that function as regulatory factors in many human diseases. Studies have shown that lncRNAs are involved in multiple cellular processes, including proliferation, apoptosis, migration, and invasion. In this report, a long non-coding RNA-ATB that is overexpressed in various tumor tissues and cell lines was investigated. Recent evidence suggests that ATB is dysfunctional in a variety of cancers, including hepatocellular carcinoma, gastric cancer (GC), colorectal cancer (CRC), breast cancer (BC), prostate cancer, renal cell carcinoma, non-small cell lung cancer (NSCLC), pancreatic cancer, osteosarcoma, and glioma. The high expression of ATB is associated with clinicopathological features of cancer patients. In addition, overexpression of lncRNA-ATB can promote tumor proliferation, migration, and invasion. LncRNA-ATB induces epithelial-mesenchymal transition (EMT) by competitively binding to miRNAs, thus promoting tumor progression. Biological functions and mechanisms of ATB in human cancers are discussed here, concluding that lncRNA-ATB may provide a new biomarker for use in diagnosis and prognosis of cancer.

## Introduction

Recent studied have shown that the number of newly diagnosed cancer patients and deaths by cancers are increasing worldwide (Siegel et al., 2015). For example, according to 2017 cancer statistics, a total of 1,688,780 new cancer cases and 600,920 cancer deaths are projected to occur in the United States alone (Siegel et al., 2017). Thus, early diagnosis and treatment of cancer is of immediate importance. As such, there is an urgent need to find new and effective biomarkers and therapeutic targeting agents.

Long non-coding RNAs (lncRNAs) belong to a class of transcriptional RNA molecules containing more than 200 nucleotides (Esteller, 2011; Zhang M. et al., 2017). LncRNAs were initially thought to be background products without any biological function resulting from transcription by RNA polymerase II (Kugel and Goodrich, 2012). However, data in the literature indicates that lncRNAs have important regulatory functions in many human diseases (Hung and Chang, 2010; Gibb et al., 2011; Ulitsky and Bartel, 2013; Wu et al., 2015; Chen et al., 2017). Particularly in cancer cells, lncRNAs affect the proliferation, growth, cycle progression, apoptosis, and migration of transformed cancer cells (Zhang et al., 2013; Li et al., 2016a,b). LncRNAs have also been noted as a tumor suppressors or oncogenes in tumors (Lian et al., 2016; Zhang J. et al., 2017). Numerous recent studies have confirmed the roles of lncRNAs in tumors, including lncRNA-PVT1, lncRNA-XIST, LINC00152, lncRNA-MALAT1, and so on (Ji et al., 2014; Fang et al., 2015; Chen et al., 2016; Huang et al., 2017; Kiang et al., 2017). LncRNAs can regulate gene expression in a variety of ways, including chromosome remodeling, transcription, and post-transcriptional treatment (Ponting et al., 2009; Batista and Chang, 2013; Li et al., 2017). For example, lncRNA-PVT1 and LINC00152 are potential oncogenes for various types of cancer (Zhou et al., 2015; Cui et al., 2016; Yu et al., 2017). lncRNA-XIST disorders and overexpression of X-linked genes may be an important factor in tumorigenesis (Weakley et al., 2011).

LncRNA-ATB is a lncRNA that is mapped to human chromosome 14:19,858,667–19,941,024. The well-characterized lncRNA-ATB has been shown as a transcript with two exons stretching over 80 kb. Overexpression of Transforming Growth Factor-β(TGF-β) leads to upregulation of lncRNA-ATB. In addition, LncRNA-ATB is overexpressed in many human cancers and affects development and progression of the disease. In this manuscript, recent ATB research is discussed and summarized regarding its function in cancer development. These studies indicate that ATB may serve as a new biomarker for the diagnosis and prognosis of cancer. Related clinicopathological features of lncRNA-ATB in cancers are presented in Table 1. Table 2 shows the effects of lncRNA in various cancers.

**Table 1:** Overexpression of lncRNA-ATB is associated with clinicopathological features.

**Cancer type**	**Clinicopathological features**	**References**
Hepatocellular carcinoma	Poor prognosis, liver cirrhosis microvascular invasion, macrovascular invasion encapsulation, PVTT tissues	Yuan et al., 2014
Gastric cancer	Poor prognosis, TNM stage vascular invasion, lymph node metastasis	Saito et al., 2015; Lei et al., 2017
Colorectal cancer	Tumor size, lymphatic invasion, poor prognosis vascular invasion, lymph node metastasis	Iguchi et al., 2015; Yue et al., 2016
Breast cancer	Trastuzumab resistance HER2-positive breast cancers	Shi et al., 2015
Prostate cancer	Poor prognosis, histological grade, biochemical recurrence preoperative PSA level, pathological stage, gleason score, lymph node metastasis, angiolymphatic invasion	Xu et al., 2016
Renal cell carcinoma	Tumor stages, histological grade, vascular invasion, lymph node metastasis, distant metastasis	Xiong et al., 2016
Non-small cell lung cancer	TNM stages, lymph node metastasis, distant metastasis tumor size, poor prognosis	Ke et al., 2017
Glioma	Tumor grades, tumor size, poor prognosis	Ma et al., 2016
Osteosarcoma	Poor prognosis, enneking stage metastasis, recurrence	Han et al., 2017
Pancreatic cancer	Lymphatic metastasis, neural invasion, prognosis neural invasion	Qu et al., 2016

*PVTT, Portal vein tumor thrombus; TNM, tumor node metastasis; HER2, human epidermal growth factor receptor 2; PSA, Prostate Specific Antigen*.

**Table 2:** The characteristics of lncRNA-ATB in various cancers.

**Cancer type**	**Effects**	**Related genes**	**References**
Hepatocellular carcinoma	EMT process, invasion colonization	miR-200s, ZEB1, ZEB2, TGF-β STAT3, IL-11	Yuan et al., 2014
Gastric cancer	EMT process, proliferation invasion, metastasis	miR-200s, miR-141-3p, ZEB TGF-β2	Saito et al., 2015; Lei et al., 2017
Colorectal cancer	EMT process, proliferation invasion	E-cadherin, ZEB1 ZO-1, N-cadherin	Iguchi et al., 2015; Yue et al., 2016
Breast cancer	EMT process, growth, invasion, apoptosis migration, trastuzumab resistance	miR-200c, ZNF-217 ZEB1	Shi et al., 2015
Prostate cancer	EMT process, proliferation	cyclin E, cyclin D1, p21, p27, PI3K/AKT, ERK	Xu et al., 2016
Renal cell carcinoma	EMT process, apoptosis, migration, Invasion, proliferation		Xiong et al., 2016
Non-small cell lung cancer	Migration, apoptosis, invasion		Ke et al., 2017
Glioma	Proliferation, growth migration, invasion	miR-200a, TGF-β2	Ma et al., 2016
Osteosarcoma	Proliferation, migration invasion	ZEB1, ZEB2 miR-200s	Han et al., 2017
Pancreatic cancer	Proliferation, migration, invasion	miR-200a	Qu et al., 2016

*EMT process, epithelial-mesenchymal transition process; ZEB1/2, zinc finger E-box binding homeobox 1/2; TGF-β, transforming growth factor β; STAT3, signal transducer and activator of transcription 3; IL-11, interleukin-11; ZNF-217, zinc finger protein 217; ZO-1, zonula occludens-1; PI3K, phosphoinositide 3-kinase; AKT, protein kinase B; ERK, extracellular signal-regulated kinase*.

## Hepatocellular carcinoma

Hepatocellular carcinoma(HCC) is one of the most common human malignancies worldwide and the third leading cause of cancer death (Au and Frenette, 2015). Diagnosis and treatment of hepatocellular carcinoma have shown great progress (Maluccio and Covey, 2012; Waghray et al., 2015). However, poor prognosis of hepatocellular carcinoma and high recurrence rate are still the main causes of death in HCC patients (Poon, 2011). Hepatocellular carcinoma metastasis includes intrahepatic and extrahepatic metastases (Budhu et al., 2006). The 5-year survival of HCC patients remains at a low rate of approximately 7% (Ilikhan et al., 2015). Invasion of HCC is affected by the cell itself and the extracellular microenvironment (Fidler, 2003). At present, the underlying molecular mechanism that affects invasion and metastasis of hepatocellular carcinoma still remains unclear. Due to the lack of understanding of the formation, progression, and recurrence of hepatocellular carcinoma, new treatments are at a standstill. One way forward involves understanding the underlying molecular mechanisms of HCC in order to search for new tumor biomarkers.

Yuan et al. (2014) identified that lncRNA-ATB expression is controlled by TGF-β and negatively associated with the miR-200 family, such as miR-429, miR-141, miR-200a, miR-200b, and miR-200c. These have been shown to promote the epithelial-mesenchymal transition (EMT) process and cell invasion by repressing ZEB1 and ZEB2 expression (Feng et al., 2014; Bracken et al., 2015; Katsura et al., 2017; Singh et al., 2017). Moreover, lncRNA-ATB could facilitate the colonization of HCC cell at the site of metastasis through interacting with, and thereby enhancing the structural stability of, IL-11 mRNA expression resulting in autocrine induction and subsequent activation of STAT3 signaling (Li and Kang, 2014). It was also confirmed that IL-11/STAT3 signaling was an indispensable pathway during the cell colonization induced by lncRNA-ATB (Yuan et al., 2014). LncRNA-ATB was significantly correlated with liver cirrhosis in HCC patients, which caused poor prognosis of HCC patients (Yuan et al., 2014). In conclusion, targeting lncRNA-ATB may contribute to the anti-metastasis therapies for HCC patients.

## Gastric cancer

Gastric cancer (GC) is a serious health problem in today's society, ranking second among all cancers (Ang and Fock, 2014). Due to the nature of GC, diagnosis is typically late in the disease, and as such, many patients miss the optimum timing for treatment. By the time of treatment onset, patients often have lymphatic metastasis and distant metastasis (Ohtsu, 2008). Therefore, prognosis of most GC patients is poor. In order to improve prognosis, new methods of early detection and diagnosis are needed (Song et al., 2013).

Saito et al. (2015) used the quantitative polymerase chain reaction (QPCR) method to detect the expression of lncRNA-ATB in GC tissue. These studies showed that lncRNA-ATB was highly expressed in GC tissue compared with normal tissue. Moreover, the expression level of lncRNA-ATB was significantly correlated with the overall post-operative survival rate of GC (Saito et al., 2015). Compared with the lncRNA-ATB low expression group, the overall survival rate of the lncRNA-ATB high expression group was lower. The data analysis showed that lncRNA-ATB was an independent prognostic factor. This study also demonstrated that TGF-β and ZEB1 levels in the high lncRNA-ATB group were higher than those of low lncRNA-ATB group, while miR-200c levels were shown to be the opposite (Saito et al., 2015). Additionally, lncRNA-ATB induced EMT through TGF-β to influence GC transfer. TGF-β receptor inhibitor SB431542 inhibited the expression of lncRNA-ATB in GC cell lines. In summary, ATB may be an independent prognostic marker for GC.

Lei et al. (2017) described the potential molecular mechanisms of lncRNA-ATB in the development of GC. Using *in vitro* experiments, it was shown that low levels of lncRNA-ATB could inhibit the proliferation, migration, and invasion of GC cells, and the knockdown of lncRNA-ATB could inhibit cell cycle arrest. Compared with the negative control (NC) group, the expression of lncRNA-ATB was negatively correlated with the expression level of miR-141-3p (Lei et al., 2017). MiR-141-3p might be a downstream effector of lncRNA-ATB in GC. TGF-β activated lncRNA-ATB and TGF-β2 was directly combined with mir-141-3p. Western blot analysis showed that lncRNA-ATB and miRNA (such as miR-141-3p) could act as competitive endogenous RNAs for TGF-β2 and promote tumor progression. In conclusion, the lncRNA-ATB through an MiR-141-3p/TGFβ2 feedback loop is helpful in the growth of GC.

## Colorectal cancer

Colorectal cancer (CRC) is the third most common cancer in the world and is considered one of the most prevalent invasive malignancies (Siegel et al., 2014). Although diagnosis and treatment of colon cancer have made significant progress, cancer recurrence and/or metastasis are still likely outcomes (Edwards et al., 2014). Therefore, the establishment of new strategies for the diagnosis and prognosis of biomarkers is an urgent task for the early diagnosis and treatment of colon cancer.

Iguchi et al. (2015) detected the expression of lncRNA-ATB in 124 patients with CRC by real-time reverse-transcription polymerase chain reaction (RT-PCR). Iguchi et al. found a significant increase in tumor size and tumor invasion depth in patients with high lncRNA-ATB expression compared with patients with low lncRNA-ATB expression. Furthermore, vascular invasion, lymphatic invasion, and lymph node metastasis were also more frequent in the lncRNA-ATB high expression group (Iguchi et al., 2015). In CRC, lncRNA-ATB overexpression was positively correlated with TNM staging, invasion depth, metastasis, and tumor size. The recurrence-free survival rate of the lncRNA-ATB overexpression group was higher than that of the low expression group. From these data it may be concluded that lncRNA-ATB might serve as a new indicator of poor prognosis in CRC patients.

Yue et al. (2016) found that the expression of lncRNA-ATB in CRC tissue and cell lines was significantly up-regulated compared with the corresponding adjacent mucosa. However, the expression level of E-cadherin in cancer tissues decreased. The decrease in E-cadherin reduced the intensity of cell adhesion within the tissue, leading to increased cell activity. This could allow cancer cells to invade surrounding tissue through the basement membrane (Kourtidis et al., 2017). Further studies showed that lncRNA-ATB was highly expressed in lymph node metastases compared with primary cancer tissue, and E-cadherin showed a low expression in lymph node metastases (Yue et al., 2016). The prognosis of patients with high lncRNA-ATB expression and low E-cadherin expression was worse. Using *in vitro* experiments, it was confirmed that the knockdown of lncRNA-ATB could up-regulate the expression of E-cadherin and ZO-1, and decrease the expression of interstitial markers such as ZEB1 and N-cadherin (Yue et al., 2016). Individual E-cadherin was not a prognostic indicator while expression of E-cadherin in colon cancer could be used as a prognostic factor. The above studies suggest that lncRNA-ATB and E-cadherin play important roles in CRC progression and metastasis. LncRNA-ATB affects the progress of colon cancer cells by inhibiting E-cadherin expression and promoting EMT action. Therefore, inhibition of lncRNA-ATB may be a new strategy for inhibition of colon cancer progression.

## Breast cancer

Breast cancer (BC) is the most abundant cancer in women and leading cause of mortality (N Cancer Genome Atlas, 2012). Distal metastasis remains the primary cause of death in BC patients. Human epidermal growth factor receptor (HER2) has become the prominent prognostic factor for BC (Rahim and O'Regan, 2017). In all subtypes of BC, HER2-positive patients show the worst prognosis. Currently, trastuzumab has been used in patients with HER2-positive BC. Trastuzumab blocks the growth of cancer cells by attaching itself to the HER2 receptor to prevent attachment of human epidermal growth factor (Vu and Claret, 2012). However, there are still many unclear molecular mechanisms involved in the resistance of trastuzumab. An understanding of the molecular mechanisms of trastuzumab resistance is vital for the treatment of BC.

Shi et al. (2015) constructed the extracorporeal SKBR-3 model to better understand trastuzumab resistance. It was found that lncRNA-ATB was significantly up-regulated in trastuzumab-resistant (TR) BC tissue and TR SKBR-3 cells. MTT assays showed that down-regulation of lncRNA-ATB significantly increased the growth inhibition and apoptosis of TR SKBR-3 cells. Highly expressed lncRNA-ATB was associated with trastuzumab resistance in BC patients. Further experiments confirmed that lncRNA-ATB up-regulation was negatively correlated with miR-200c levels and positively correlated with ZEB1 and ZNF217 levels in TR BC tissue and TR SKBR-3 cells (Shi et al., 2015). Taken together, lncRNA-ATB acts as a ceRNA which competes with miR-200c in TR SKBR-3 cells, then up-regulates ZEB1 and ZNF-217 and induces EMT to promote trastuzumab resistance and an invasion-metastasis cascade in BC. Therefore, lncRNA-ATB is one of the factors affecting the prognosis of patients with BC and trastuzumab resistance.

## Urologic neoplasms

Urologic neoplasms are one of the most prevalent malignancies in the world, including prostate cancer, renal cell carcinoma, and bladder cancer. Prostate cancer is the most commonly diagnosed malignancy in men worldwide and the second leading cause of cancer deaths in the United States (Scher et al., 2015). Renal cell carcinoma is one of the most malignant urinary cancers (Rini et al., 2008). Although the diagnosis and treatment of urological neoplasms have been developed greatly, many patients have been diagnosed with distant metastasis. Moreover, the 5-year survival rate of cancer patients is low. Therefore, it is a critical task to find an effective biomarker for the prognosis of these tumors.

Toward this end, Xu et al. (2016) demonstrated the up-regulation of lncRNA-ATB expression in prostate cancer by QPCR experiments. The Kaplan-Meier curves showed that prostate cancer patients with a high expression of lncRNA-ATB had a worse biochemical recurrence-free survival rate than patients with a low expression of lncRNA-ATB. The knockdown of lncRNA-ATB significantly attenuated the proliferation rate in prostate cell lines, but the forced overexpression of lncRNA-ATB was effective in promoting proliferation. Xu et al. found that lncRNA-ATB knockout significantly increased the expression levels of E-cadherin and ZO-1 (Xu et al., 2016). In addition, the high expression of lncRNA-ATB simulated the phosphorylation levels of Akt and ERK in PC-3 and DU-145 prostate cell lines. Additionally, the ERK and PI3K/AKT signaling pathways stimulated the expression levels of ZEB1 and ZNF217 proteins, thereby promoting EMT. Their study suggested that lncRNA-ATB might be a potential predictor of clinical prognosis in prostate cancer.

Xiong et al. (2016) found lncRNA-ATB was overexpressed in renal cell carcinoma and cell lines. The expression level of lncRNA-ATB was correlated with tumor staging, histological classification, vascular infiltration, lymph node metastasis, and distant metastasis. Using *in vitro* experiments, it was confirmed that lncRNA-ATB knockout could inhibit the proliferation, migration, and invasion of renal cell carcinoma cells and promote apoptosis (Xiong et al., 2016). Furthermore, knockdown of lncRNA-ATB resulted in up-regulation of E-cadherin expression and down-regulation of N-cadherin and vimentin expression. LncRNA-ATB could induce EMT in renal cell carcinoma. In conclusion, lncRNA-ATB may be a new prognostic biomarker of renal cell carcinoma.

## Non-small cell lung cancer

It is widely accepted that lung cancer is one of the leading causes of cancer death in the world (Siegel et al., 2015); more than 85% of the cases are non-small cell lung cancer (NSCLC) (Chen et al., 2014). Surgery and chemotherapy are the main therapeutic methods for lung cancer; however, the 5-year overall survival of NSCLC patients is very low (Tarasevych et al., 2014). The molecular mechanisms involved in the development of NSCLC remains unclear. Therefore, prognostic biomarkers of NSCLC are urgently needed (Yu et al., 2015).

Ke et al. (2017) found that the expression levels of lncRNA-ATB in NSCLC tissue was significantly higher than that in normal tissue. In this study, 84 NSCLC patients were followed. It was found that patients with high lncRNA-ATB expression and low lncRNA-ATB expression had a cumulative survival rate of 32.6 and 68.4%, respectively (Ke et al., 2017). It was shown in *in vitro* experiments that suppression of lncRNA-ATB promoted cell apoptosis and inhibited cell migration and invasion of A549 cells. Therefore, lncRNA-ATB has the potential to become a prognostic marker for NSCLC.

## Other cancers

Recent studies have shown that lncRNA-ATB can also affect the development and prognosis of gliomas, osteosarcoma, and pancreatic cancer (Ma et al., 2016; Qu et al., 2016; Han et al., 2017). Experiments have shown that lncRNA-ATB can promote cell proliferation, migration, and invasion, and inhibit cell apoptosis in these cancers.

Glioma is one of the most common primary malignant tumors in brain. Ma et al. (2016) found that lncRNA-ATB was highly expressed in glioma tissue and cell lines compared to normal brain tissue. Moreover, the high expression of lncRNA-ATB in glioma patients was negatively correlated with overall survival. Studies using *in vitro* functional experiments showed that the inhibition of lncRNA-ATB expression could significantly inhibit glioma cell proliferation, migration, and invasion (Ma et al., 2016). In addition, lncRNA-ATB could act as a ceRNA to regulate the target TGF-β2 of mir-200a. The results showed that lncRNA-ATB could inhibit mir-200a and promote TGF-β2 in glioma cells. Thus, lncRNA-ATB may be a new target for treating human gliomas and improving prognosis.

Han et al. (2017) confirmed that the expression of lncRNA-ATB increased in osteosarcoma tissues. The knockdown of lncRNA-ATB significantly inhibited the proliferation, migration, and invasion of osteosarcoma cells (Han et al., 2017). Additionally, lncRNA-ATB affected the proliferation, migration, and invasion of osteosarcoma cells *in vitro* and the growth of osteosarcoma *in vivo* by inhibition of miR-200s. In summary, lncRNA-ATB may be a potential therapeutic target for osteosarcoma.

Qu et al. (2016) showed the opposite results in pancreatic cancer. In this study it was found that lncRNA-ATB showed a low expression in pancreatic cancer tissue and cell lines. Low expression levels of lncRNA-ATB were significantly correlated with lymph node metastasis, nerve invasion, and clinical stage in pancreatic cancer (Qu et al., 2016). Moreover, the overall survival of patients with low expressions of lncRNA-ATB was worse. In summary, the Qu et al. study showed that the down-regulation of lncRNA-ATB was an independent prognostic factor for pancreatic cancer patients. Thus, lncRNA-ATB may be a potential prognostic marker for pancreatic cancer (Garrido-Laguna and Hidalgo, 2015).

## Discussion

**Figure 1:**
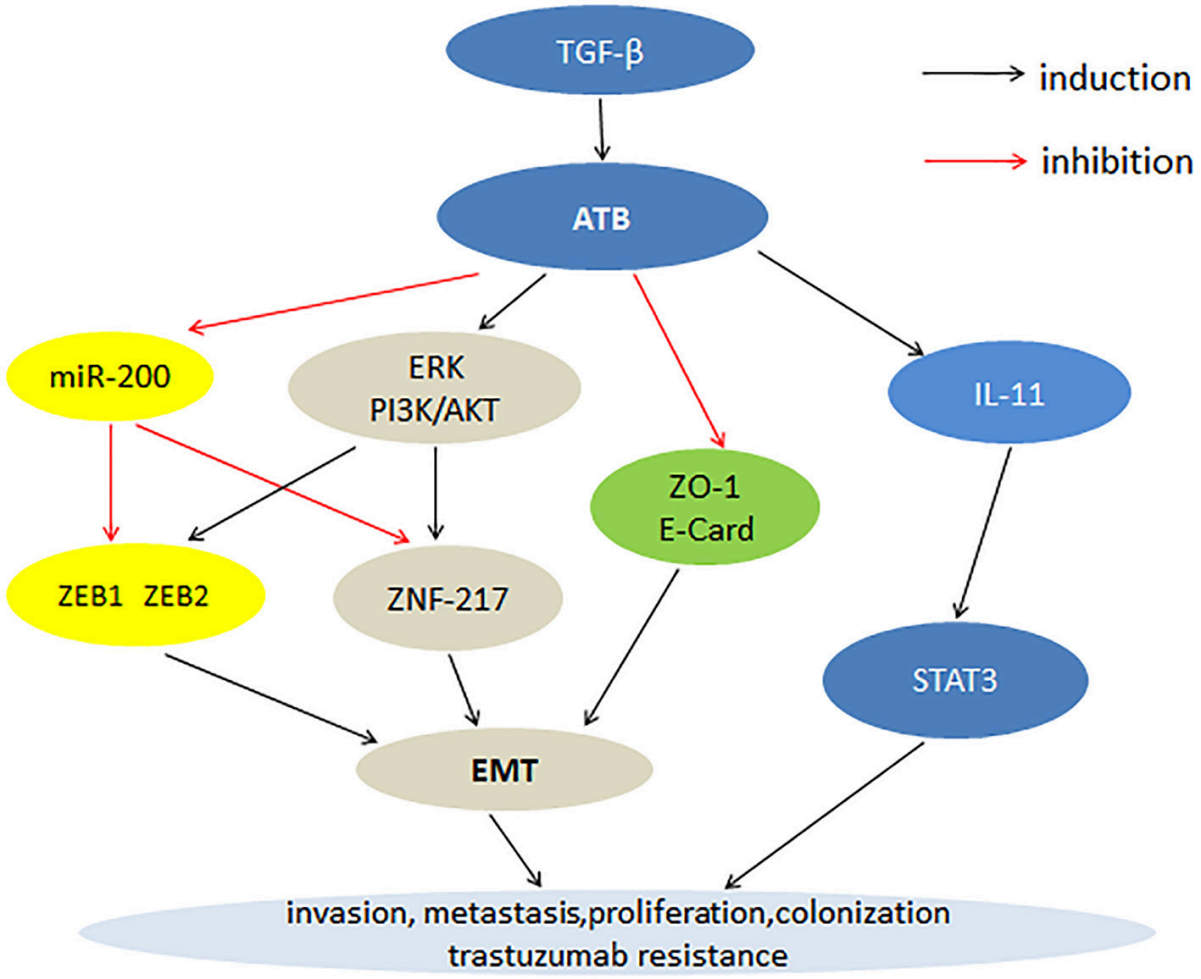
Mechanisms of lncRNA-ATB involvement in cancer progression.

Cancer is a major health issue often accompanied by genetic mutation. At present, the treatment and prognosis of cancer patients remains unsatisfactory. lncRNA is a class of transcribed RNA molecules. LncRNA are involved in genetic regulation, transcription and post-transcriptional regulation, and play important roles in cancer development. The functional significance of various lncRNAs in cancer has been reported, such as lncRNA-PVT1, lncRNA-XIST, and LINC00152. LncRNA-ATB as a carcinogenic factor is overexpressed in a variety of cancers, including hepatocellular carcinoma, GC, CRC, BC, prostate cancer, renal cell carcinoma, NSCLC, pancreatic cancer, osteosarcoma, and glioma. In tumor tissues, high expression level of lncRNA-ATB is closely related to clinicopathological features such as TNM stage, tumor size, histological grade, distant metastasis, lymphatic metastasis and poor prognosis. Cell experiments have shown that the overexpression of lncRNA-ATB can promote cancer cell proliferation, migration, invasion, and inhibition of apoptosis. Fan et al. (2017) showed that lncRNA-ATB was also a useful prognostic biomarker for human cancer. Therefore, lncRNA-ATB can be used as a new biomarker to treat cancer. The current research has shown that the mechanism by which lncRNA-ATB promotes tumor development involves multiple steps. First, lncRNA-ATB inhibits the expression of ZEB1, ZEB2, and ZNF-217 through competitive binding with the miR-200 family, thereby promoting EMT progression and cell invasion. Second, lncRNA-ATB stimulates the expression of ZEB1 and ZNF217 proteins by activating the ERK and PI3K/AKT signaling pathways, thereby promoting the EMT process. Third, the high expression of lncRNA-ATB inhibits the expression of E-cadherin and ZO-1, leading to the EMT process. Lastly, lncRNA-ATB activates the autocrine induction of IL-11 by binding to IL-11, and then activates STAT3 signaling to promote cancer cell colonization (Figure 1).

At present, lncRNA-ATB research is still in the preliminary stages, such that the precise functional role of lncRNA-ATB remains unclear. Thus, the detailed molecular mechanism of lncRNA-ATB requires further study. The use of lncRNA-ATB as a biomarker for the diagnosis and treatment of tumors needs to address several challenges. First, the number of cancer patients currently being studied by lncRNA-ATB is limited, leading to the unconvincing clinical results due to low sample size. Second, no specific index of lncRNA-ATB concentration in the blood of cancer patients and healthy people has been established, which obstructs the clinical study of lncRNA-ATB. Third, the interaction of lncRNA-ATB with the most common cancer pathogenesis (chromosomal abnormalities, histone modifications, DNA methylation) is unclear. Finally, lncRNA-ATB uses a variety of mechanisms to exert its function, but the process by which lncRNA-ATB controls these mechanisms in different types of cancer cells is unclear. Addressing these challenges will contribute to an in-depth understanding and use of lncRNA-ATB. At present, precise medical treatments, such as molecular targeted therapy, have gained increased attention and research. Targeted therapies have the advantage of precise specificity and low systemic toxicity. In future studies, lncRNA-ATB may serve as a new molecular target for the treatment of cancer.

In conclusion, lncRNA-ATB expression has been shown to be upregulated in a variety of human cancers serving as a potent carcinogen. LncRNA-ATB can be used as a new biomarker for the diagnosis and prognosis of cancer. At present, research on lncRNA-ATB is still the beginning; however, in time it is likely that lncRNA-ATB will be used as a molecular targeted drug therapy for clinical use.

## Author contributions

YL: Conceived and designed the work; HX, and JL: Drafted the manuscript; FZ, and YZ: Revised the manuscript; WH: Approved the final version.

### Conflict of interest statement

The authors declare that the research was conducted in the absence of any commercial or financial relationships that could be construed as a potential conflict of interest.
